# Human Health Safety of Animal Feeds Workshop

**DOI:** 10.3201/eid1012.040987

**Published:** 2004-12

**Authors:** Vrinda N. Nargund

**Affiliations:** * Centers for Disease Control and Prevention, Atlanta, Georgia, USA

**Keywords:** animal feeds, bacteria, food contamination, conference summary

Approximately 150 scientists attended the "Human Health Safety of Animal Feeds" workshop at the Centers for Disease Control and Prevention (CDC) on January 23, 2004, to discuss issues pertaining to *Salmonella*-contaminated animal feed and their impact on public health. The workshop followed an article published in Clinical Infectious Diseases, which provided three recommendations to reduce human foodborne disease caused by *Salmonella*-contaminated animal feed (*1*). The first recommendation stressed the need for microbial contamination surveillance to determine how feed contaminants, particularly *Salmonella*, pass through the food chain. The second recommendation was to establish hazard analysis and critical control point programs to minimize *Salmonella* contamination by identifying and controlling sources of feed contamination. The third recommendation was to implement the *Salmonella*-negative standard in the feed industry. The purpose of the workshop was to elicit discussion on these and other recommendations concerning the human health safety of animal feed.

A variety of organizations were represented at the workshop, including international government agencies, the United States Department of Agriculture (USDA), the United States Food and Drug Administration (FDA), and consumer groups. Speakers offered perspectives on bacterial contamination of animal feed, including examples of human illnesses traced to *Salmonella*-contaminated feed, and data showing how contaminated animal feed contributes to human foodborne illness.

The opening plenary session focused on international experiences in controlling *Salmonella* in animal feed. Officials from the National Veterinary Institute of Sweden and the Norwegian Agriculture Inspection Service gave an overview of the control measures implemented in Sweden and Norway to ensure *Salmonella*-negative animal feed. Norway and Sweden have extensive surveillance programs for *Salmonella* control in animal feed. The measures implemented in Norway and Sweden are important contributing factors to the virtual absence of *Salmonella* in the food supply in their countries.

Several U.S. government agencies, including CDC; USDA; and the National Institutes of Health, National Institute of Allergy and Infectious Diseases, presented research findings at the workshop. Presentations included results from animal feed commodity studies that look at the factors contributing to microbial pathogens, mycotoxins, and chemical residues in animal feed. Researchers from FDA and Washington State University also provided data indicating that contaminated animal feed continues to be a source of *Salmonella* in food animals.

Further studies are necessary to document the precise contribution of contaminated animal feed to human illness. Nevertheless, some presentations suggest that practical interventions are available to reduce the prevalence of *Salmonella*-contaminated animal feed. Collaboration among all groups was stressed as a useful measure in controlling contaminated animal feed in the future.

A compact disk, including all of the presentations, agenda, and list of participants from the workshop, is available online at http://www.cdc.gov/narms/mce/animalfeeds.htm.
